# Calculating second derivatives of population growth rates for ecology and evolution

**DOI:** 10.1111/2041-210X.12179

**Published:** 2014-05-19

**Authors:** Esther Shyu, Hal Caswell

**Affiliations:** 1 Biology Department MS-34, Woods Hole Oceanographic InstitutionWoods Hole, MA, 02543, USA; 2 Institute for Biodiversity and Ecosystem Dynamics, University of AmsterdamAmsterdam, The Netherlands

**Keywords:** eigenvalues, Hessian matrix, invasion exponent, matrix population models, net reproductive rate, sensitivity analysis

## Abstract

**1.** Second derivatives of the population growth rate measure the curvature of its response to demographic, physiological or environmental parameters. The second derivatives quantify the response of sensitivity results to perturbations, provide a classification of types of selection and provide one way to calculate sensitivities of the stochastic growth rate.

**2.** Using matrix calculus, we derive the second derivatives of three population growth rate measures: the discrete-time growth rate λ, the continuous-time growth rate *r* = log λ and the net reproductive rate *R*_0_, which measures per-generation growth.

**3.** We present a suite of formulae for the second derivatives of each growth rate and show how to compute these derivatives with respect to projection matrix entries and to lower-level parameters affecting those matrix entries.

**4.** We also illustrate several ecological and evolutionary applications for these second derivative calculations with a case study for the tropical herb *Calathea ovandensis*.

## Introduction

Using matrix population models, ecological indices can be calculated as functions of vital rates such as survival or fertility. Measures of population growth rate, including the discrete-time growth rate λ, the continuous-time growth rate *r* = log λ and the net reproductive rate *R*_0_, are of particular interest. The discrete-time population growth rate λ is given by the dominant eigenvalue of the population projection matrix. Sensitivities (first partial derivatives) of λ with respect to relevant parameters quantify how population growth responds to vital rate perturbations. These first derivatives are used to project the effects of vital rate changes due to environmental or management perturbations, uncertainty in parameter estimates and phenotypic evolution (i.e. with λ as a fitness measure, the sensitivity of λ with respect to a parameter is the selection gradient on that parameter) (Caswell 2001).

### Applications of second derivatives of growth rates

The second derivatives of growth rates have applications in both ecology (e.g. assessing and improving recommendations from sensitivity analysis, approximating the sensitivities of stochastic growth rates) and evolution (e.g. characterizing nonlinear selection gradients and evolutionary equilibria). Several of these applications are summarized in Table 1 and described in the following sections.

#### Second-order sensitivity analysis and growth rate estimation

The sensitivity of growth rate provides insight into the population response to parameter perturbations. However, such perturbations also affect the sensitivity itself, that is, sensitivity is ‘situational’ (Stearns 1992). These second-order effects are quantified by the sensitivity, with respect to a parameter θ_*j*_, of the sensitivity of λ to another parameter θ_*i*_, that is, by the second derivatives 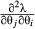. The sensitivity of the elasticity of growth rate to parameters similarly depends on second derivatives (Caswell, [Bibr b4],[Bibr b5]).

**Table 1:** Potential applications for the pure and mixed second derivatives of λ. Analogous interpretations apply to *r* or *R*_0_ as alternative measures of growth or fitness

Second derivative	Sign	Interpretations
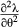	=0	Sensitivity of λ to θ is independent of θ Linear selection on trait θ
	>0	Sensitivity of λ to θ increases with θ Convex selection on trait θ Evolutionarily unstable singular strategy
	<0	Sensitivity of λ to θ decreases with increases in θ Concave selection on trait θ Evolutionarily stable singular strategy
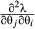	>0	Sensitivity of λ to θ_*i*_ increases with θ_*j*_ Selection to increase correlation between traits θ_*j*_ and θ_*i*_
	<0	Sensitivity of λ to θ_*i*_ decreases with increases in θ_*j*_ Selection to decrease correlation between traits θ_*j*_ and θ_*i*_
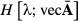	N/A	Used to calculate sensitivity of the stochastic growth rate λ_*s*_

In conservation applications, attention is often focused on the vital rates to which population growth is particularly sensitive or elastic; these first-order results may change depending on parameter perturbations. First derivatives also provide a linear, first-order approximation to the response of the growth rate to changes in parameters. The linear approximation is guaranteed to be accurate for sufficiently small perturbations and is often very accurate even for quite large perturbations (Caswell 2001). If the response of λ to θ is nonlinear, it is tempting to use a second-order approximation for Δλ:


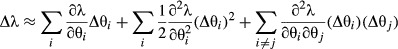
eqn 1

**Fig. 1:**
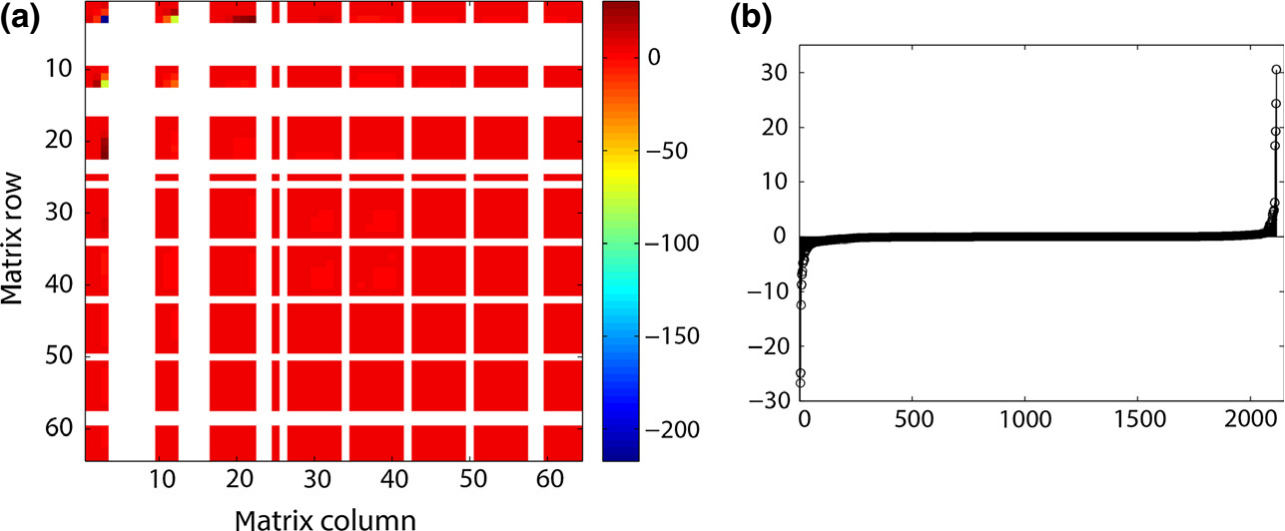
(a) The Hessian matrix *H*[λ; vec**A**], giving the second derivatives of λ with respect to the entries of the projection matrix **A**, for *C. ovandensis*. Entries corresponding to fixed zeros (unobserved transitions) in the matrix (59) are omitted. (b) The entries of the Hessian matrix in 1a, sorted in ascending order. The derivatives 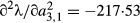 and ∂^2^λ/∂*a*_3,1_∂*a*_4,2_ = −75·64 are omitted due to their magnitude.

We caution that although this may, in some cases, provide a more accurate calculation, this is not guaranteed. As shown in Fig. 1 of Carslake, Townley & Hodgson (2008), for example, adding the second-order terms may actually reduce the accuracy of the approximation.

#### Characterizing nonlinear selection processes

The second derivatives of fitness with respect to trait values have consequences for selection. The first derivatives of fitness are selection gradients (Lande 1982). When fitness is a linear function of a trait, its second derivatives are zero, and there is selection to shift the trait's mean value. When fitness is a nonlinear function of a trait, its second derivatives are nonzero and provide additional information on how selection affects the trait's higher moments (Lande & Arnold 1983, Phillips & Arnold 1989, Brodie, Moore & Janzen 1995). Such nonlinear selection can be classified as concave or convex depending on whether the second derivatives are negative or positive.

One can classify a selection process as linear, concave or convex using quadratic selection gradients, the local second derivatives of fitness with respect to trait value (Phillips & Arnold 1989). If fitness is measured as λ, these quadratic selection gradients are equivalent to ∂^2^λ/∂θ^2^, the pure second derivatives of λ with respect to trait θ (e.g. the second derivatives with respect to stage-specific survival in *C. ovandensis*, as shown in Fig. 3a). Concave, linear and convex selection correspond to negative, zero and positive second derivatives, respectively.

Concave selection reduces the variance in the trait, and convex selection increases it; Lande & Arnold (1983, p.1216) equate this to a more sophisticated version of the concepts of stabilizing and disruptive selection. Brodie, Moore & Janzen (1995) provide further analysis of the curvature of the fitness surface and its effects on selection.

Selection operating on pairs of traits is said to be correlational if the cross second derivatives are nonzero. Thus, if the pure second derivatives of two different traits, θ_*i*_ and θ_*j*_, are both nonzero, their mixed second derivative ∂^2^λ/∂θ_*j*_∂θ_*i*_ is a measure of correlational selection. If ∂^2^λ/∂θ_*j*_∂θ_*i*_<0, there is selection to decrease the phenotypic correlation between the two traits; if ∂^2^λ/∂θ_*j*_∂θ_*i*_>0, there is selection to increase their correlation. The concepts of nonlinear selection are powerful, but require the second derivatives of fitness to be applied.

#### Stability of evolutionary singular strategies

Second derivatives play a role in adaptive dynamic analyses. Evolutionary singular strategies (SSs) are phenotypes for which the selection gradient is locally zero (e.g. Geritz *et al*. 1998). SSs are classified as stable, attracting or repelling, and by whether they can invade or coexist with other nearby phenotypes (Geritz *et al*. 1998, Diekmann 2004, Waxman & Gavrilets 2005, Doebeli 2011).

These classifications depend on the local second derivatives of invasion fitness, the growth rate of a rare mutant in an equilibrium resident environment. For example, the second derivative of the mutant growth rate λ to the mutant trait *y* determines whether a SS is evolutionarily stable (∂^2^λ/∂*y*^2^<0) or evolutionarily unstable (∂^2^λ/∂*y*^2^>0). Evolutionarily stable strategies, once established, are unbeatable phenotypes against which no nearby mutants can increase under selection and are thus long-term evolutionary endpoints. Evolutionarily unstable strategies, on the other hand, are branching points open to phenotypic divergence and may ultimately become sources of sympatric speciation (Geritz *et al*. 1998).

#### Sensitivity of the stochastic growth rate

Second derivatives provide a way to calculate the sensitivity of the stochastic growth rate in some cases. The stochastic growth rate is


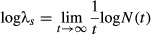
eqn 2

where *N*(*t*) is the population size at time *t*. Tuljapurkar (1982) derived a small-noise approximation for log λ_*s*_ in the absence of temporal autocorrelation. As shown by Caswell (2001 Section 14.3.6), this approximation can be written in terms of the first derivatives of λ, the dominant eigenvalue of the mean projection matrix 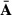. Thus, the derivatives of this approximation can be written in terms of the second derivatives of that eigenvalue (Caswell 2001, Section 14.3.6). We discuss this application further in the section ‘Sensitivity analysis of stochastic growth rates’.

### Calculating second derivatives of growth rates

The second derivatives of λ with respect to matrix elements were introduced by Caswell (1996); see also Caswell (2001, Section 9.7). However, these calculations are awkward and error-prone, because they involve all the eigenvalues and eigenvectors of the projection matrix. McCarthy, Townley & Hodgson (2008) introduced an alternative approach for calculating the second derivatives of eigenvalues (they call them ‘second-order sensitivities’) based on transfer functions, partially to avoid the calculation of all the eigenvectors. However, they consider only rank-one perturbations of a subset of the matrix elements, excluding fertilities, and their calculations are perhaps equally difficult.

Here, we reformulate the second derivative calculations using matrix calculus, providing easily computable results. We extend previous results by including not only second derivatives with respect to matrix elements, but also those with respect to any lower-level parameters that may affect the matrix elements, and by presenting the second derivatives of the continuous-time invasion exponent *r* and the net reproductive rate *R*_0_.

The key to our approach is that the calculation of first derivatives using matrix calculus yields a particular expression, the differentiation of which leads directly to the second derivatives. Second derivatives are easily computed by this method in any matrix-oriented language, such as Matlab or R. Although we consider only the second derivatives of population growth rates, our approach extends naturally to other scalar-dependent variables.

In the section ‘A case study: *Calathea ovandensis*’, we present an example of the calculation of second derivatives in a case study of the tropical herb *Calathea ovandensis*.

### Notation

Matrices are denoted by upper-case boldface letters (e.g. **A**) and vectors by lower-case boldface letters (e.g. **w**); unless otherwise indicated, all vectors are column vectors. Transposes of matrices and vectors are indicated by the superscript 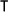. The matrix **I**_*n*_ is the *n*×*n* identity matrix, the vector **e** is a vector of ones, and **e**_1_ is a vector with 1 as its first entry and zeros elsewhere. The matrix **K**_*m*,*n*_ is a *mn*×*mn* commutation matrix (vec-permutation matrix) (Magnus & Neudecker 1979, Henderson & Searle 1981), which can be calculated using the Matlab function provided in Appendix S1-D. The expression diag(**x**) indicates the square matrix with **x** on the diagonal and zeros elsewhere.

The Kronecker product is denoted by **X**⊗**Y** and the Hadamard (element-by-element) product by **X**∘**Y**. The vec operator (e.g. vec**A**) stacks the columns of a matrix into a single vector. For convenience, we will write (vec**A**)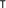 as vec
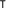
**A**. We will make frequent use of Roth's theorem (Roth 1934), which states that for any matrices **X**, **Y** and **Z**:


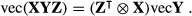
eqn 3

## Matrix calculus

### Matrix calculus notation

Matrix calculus is a system for manipulating vectors and matrices in multivariable calculus and simplifies partial derivative calculations by allowing the differentiation of scalar, vector or matrix functions with respect to scalar, vector or matrix arguments. While there are multiple matrix calculus notations, we will use the system of Magnus & Neudecker (1999). For a more detailed introduction to these methods in an ecological context, see Appendix 1 of Caswell (2007).

The first derivative of a *m*×1 vector **y** with respect to a *n*×1 vector **x** is defined to be the *m*×*n* Jacobian matrix


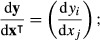
eqn 4

that is, a matrix whose (*i*,*j*) entry is the derivative of *y*_*i*_ with respect to *x*_*j*_. We will also write this as an operator *D*[**y**;**x**]; the first argument of *D* is the vector-valued function **y** to be differentiated, and the second argument is the vector-valued variable **x** with respect to which differentation is carried out. Thus,


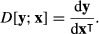
eqn 5

As in the scalar case, second derivatives are obtained by differentiating first derivatives. If we consider a scalar-valued function *y*(**x)** of a vector-valued argument **x**, the matrix of second derivatives (the Hessian matrix) is given by the operator


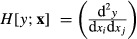
eqn 6


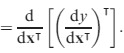
eqn 7

The matrix of second derivatives of a vector-valued function **y**(**x**), where **y** has dimensions *m*×1, is obtained by stacking the Hessian matrices for each of the elements of **y**; that is,


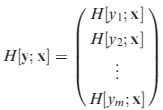
eqn 8


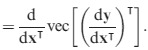
eqn 9

These first and second derivative definitions are written in terms of vector-valued functions and arguments. When matrices appear, they are transformed into vectors using the vec operator, which stacks the columns of the matrix into a column vector. Thus, the first and second derivatives of λ with respect to the entries of the matrix **A** would be written, respectively, as *D*[λ;vec**A**] and *H*[λ;vec**A**].

### The identification theorems

Magnus & Neudecker (1985, 1999) showed how to obtain first and second derivatives from the differentials of functions. Their ’first identification theorem’ showed that


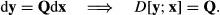
eqn 10

That is, if an expression of the form d**y**=**Q**d**x** can be obtained, then the Jacobian matrix of first derivatives is given by **Q**.

The ‘second identification theorem’ does the same for the Hessian matrix of second derivatives, showing that


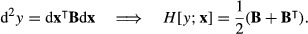
eqn 11

Thus, our goal will be to find expressions of the form d^2^*y* = d**x**
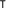
**B**d**x**, where *y* is a measure of population growth rate and **x** represents either matrix entries or lower-level parameters; the matrix **B** will then provide the Hessian matrix using (11). The key to our approach is to begin with the expression (10) for the first differential, differentiate it to obtain the second differential and manipulate the results to obtain a matrix **B** in the form of (11).

## Second derivatives of growth rates

We now apply the identification theorems to three measures of population growth rate, the discrete-time growth rate λ, the continuous-time growth rate *r* = log λ and the net reproductive rate *R*_0_.

### Second derivatives of the discrete-time growth rate λ

#### Second derivatives of λ with respect to matrix entries: *H* [λ;vec**A**]

We assume a population projection matrix **A** of dimension *n*×*n*. The discrete-time growth rate λ is the dominant eigenvalue of **A**. To derive *H* [λ;vec**A**], we begin with an expression of the form (10) for the first differential of λ. As shown in Caswell (2010),


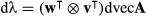
eqn 12

where **w** and **v** are the right and left eigenvectors of **A** corresponding to λ, scaled so that


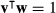
eqn 13


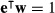
eqn 14

where **e** is a *n*×1 vector of ones.

Differentiate (12) to obtain the second differential


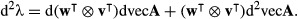
eqn 15

Because we are calculating second derivatives with respect to **A**, the second term will drop out because d^2^vec**A** = 0 (Magnus & Neudecker 1999). Apply the vec operator to obtain


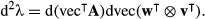
eqn 16

The differential of vec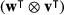 is


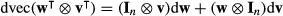
eqn 17

(Magnus & Neudecker 1999). Substituting (17) into (16) gives


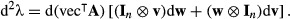
eqn 18

By the chain rule,


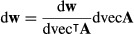
eqn 19


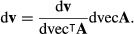
eqn 20

The first derivatives of **w** and **v**, subject to (13) and (14), are given in Caswell (2008) and H. Caswell and Y. Vindenes (unpublished data), respectively, as:


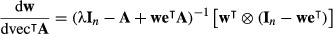
eqn 21


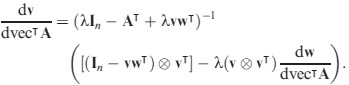
eqn 22

Substituting (19) and (20) into (18) gives


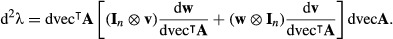
eqn 23

This is of the form


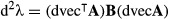
eqn 24

and hence


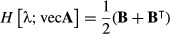
eqn 25a

where


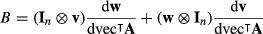
eqn 25b

and the first derivatives of **w** and **v** are given by (21) and (22).

#### Second derivatives of λ with respect to lower-level parameters: *H* [λ; ***θ***]

Because many life-history traits and environmental factors affect multiple life cycle transitions, the entries of **A** are usually functions of lower-level parameters. The first derivatives with respect to lower-level parameters are calculated with the chain rule. To calculate the second derivatives of λ with respect to a *s*×1 vector ***θ*** of lower-level parameters, we must develop a chain rule for the Hessian.

To do so, we begin with the first differential of λ in (12) and differentiate to obtain the second differential (12). Because we are calculating second derivatives with respect to ***θ*** rather than **A**, d^2^vec**A** is no longer zero. By the chain rule,


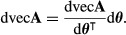
eqn 26

Differentiate (26) to obtain


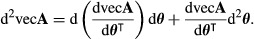
eqn 27

Because d^2^***θ*** = 0, the second term drops out.

Substituting (26) and (27) into the expression for the second differential in (12) yields


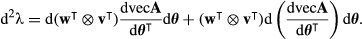
eqn 28

To simplify this expression, define


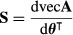
eqn 29


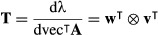
eqn 30

in terms of which (28) can be rewritten as


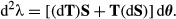
eqn 31

Then apply the vec operator and Roth's theorem (3) to obtain


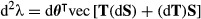
eqn 32


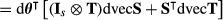
eqn 33


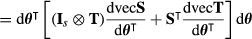
eqn 34

where, as shown by (A-10) and (A-13) in Appendix S1-A,


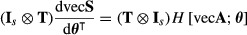
eqn 35


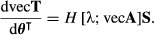
eqn 36

The expression (34) is of the form


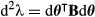
eqn 37

and hence by the second identification theorem (11),


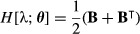
eqn 38a

where


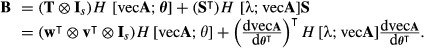
eqn 38b

The first and second derivatives of **A** with respect to **θ**, which appear in 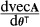 and *H* [vec**A**; ***θ***], respectively, can be evaluated by hand or by using a symbolic math program. This result is in agreement with the Hessian chain rule derived in a different way by Magnus & Neudecker 1999, p. 125).

These results can be used to parameterize constraints or covariation among traits. As a simple example, suppose that survival and fertility are constrained to covary as *F*_*i*_ = *cP*_*i*_, and one wants the total second derivative including this constraint. This is obtained by defining a lower-level parameter θ, setting *F*_*i*_ = θ and *P*_*i*_ = *c*θ and calculating *H* [λ;θ].

### Second derivatives of the invasion exponent *r*: *H*[*r*;vec**A**] and *H*[*r*;***θ***]

The population growth rate in continuous time is the invasion exponent *r* = log λ. By the definition of the Hessian in (7), the Hessian of *r* with respect to **A** is


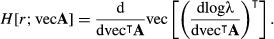
eqn 39

We insert the first derivative of log λ,


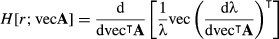
eqn 40

and then apply the product rule to obtain


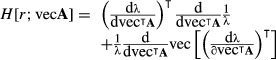
eqn 41

which simplifies to


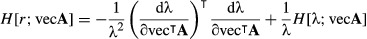
eqn 42


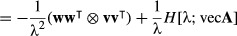
eqn 43

where *H*[λ;vec**A**] is given by (25).

Replacing vec**A** in (42) with a parameter vector ***θ*** gives the Hessian


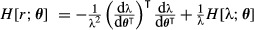
eqn 44


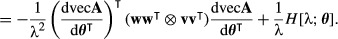
eqn 45

The derivatives 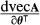 can be calculated by hand or with a symbolic math program, and *H* [λ;***θ***] can be obtained from (38).

### Second derivatives of the net reproductive rate *R*_0_

The net reproductive rate *R*_0_ measures the population growth rate per generation and is used as an alternative fitness measure to *r* under some special conditions (Pásztor, Meszéna & Kidsi 1996, Brommer 2000). If **A** is decomposed into transition and fertility matrices, **A** = **U**+**F**, then *R*_0_ is the dominant eigenvalue of the next generation matrix **R** = **FN** (Cushing & Zhou 1994), where **N** is the fundamental matrix:


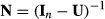
eqn 46

The (*i*,*j*) entry of **N** gives the expected number of visits to stage *i* for an individual starting in stage *j*. The (*i*,*j*) entry of **R** gives the expected lifetime production of stage *i* offspring by an individual starting in stage *j*.

Because *R*_0_ is an eigenvalue, our results for *H*[λ;vec**A**] and *H*[λ;**θ**] can be applied to find its second derivatives, but with **R** taking the place of matrix **A**. The resulting expressions are more complicated than the corresponding expressions for λ, because parameters can affect *R*_0_ through **U**, **F** or both. In the important special case where only a single type of offspring is produced (suppose it is numbered as stage 1), then **R** is an upper triangular matrix and *R*_0_ is its (1,1) entry; in this case, eigenvalue calculations are not necessary.

We defer the fully general calculation of *H*[*R*_0_;***θ***] to Appendix S1-C and show results here for two useful special cases: the second derivatives with respect to the entries of the transition matrix **U** and with respect to the entries of the fertility matrix **F**. We consider both single and multiple types of offspring.

If we apply (38) to the case of *R*_0_, replacing vec**A** with vec**R**, we obtain


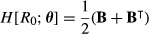
eqn 47a

where


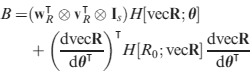
eqn 47b

and **w**_*R*_ and **v**_*R*_ are the right and left eigenvectors of **R**.

To evaluate (47), we must calculate the second derivatives of *R*_0_ with respect to **R**, and the first and second derivatives of **R** with respect to ***θ***. For the former, the Hessian *H*[*R*_0_;vec**R**] is given by (25), using the dominant eigenvalues and eigenvectors of **R** rather than those of **A**. For the latter, we will consider the derivatives of **R** with respect to **U** and **F** in turn. The derivatives of **R** with respect to general parameters ***θ*** are shown in Appendix S1-B.

#### Second derivatives of *R*_0_ *to the transition matrix: H* [*R*_0_; vec**U**]

The second derivatives of *R*_0_ with respect to the entries of the transition matrix **U** require the first and second derivatives of **R** with respect to **U**. The first derivatives are obtained by differentiating **R** = **FN**, applying the vec operator and noting that dvec**N** = (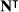
⊗**N**)dvec**U** (Caswell 2006, 2009), to obtain


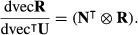
eqn 48

The second derivatives of **R** are obtained from the definition of the Hessian matrix (9):


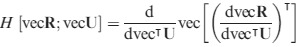
eqn 49


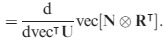
eqn 50

The derivative of vec (**N**⊗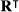) is given by a result of Magnus & Neudecker (1985, Theorem 11; 1999, p. 209); for a *m*×*n* matrix **X** and a *p*×*q* matrix **Y**,


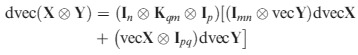
eqn 51

Thus, (50) can be rewritten as


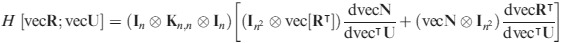
eqn 52


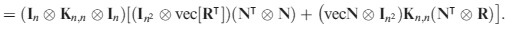
eqn 53

As a result,


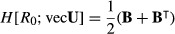
eqn 54a

where


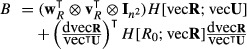
eqn 54b

where 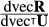 is given by (48) and *H* [vec**R**; vec**U**] is given by (53).

#### Second derivatives of *R*_0_ *to the fertility matrix*: *H* [*R*_0_; vec**F**]

Now consider the second derivatives of *R*_0_ with respect to the entries of the fertility matrix **F**. Differentiating **R** = **FN** with respect to **F** yields the first derivatives


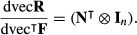
eqn 55

The second derivatives of **R** are given by the Hessian matrix


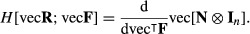
eqn 56

However, because **N** depends only on **U**, and not on **F**, this is a zero matrix.

Substituting (55) and (56) into (47) gives


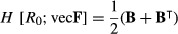
eqn 57a

where


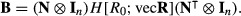
eqn 57b

#### Single type of offspring

In the common case where there is only one type of offspring (Appendix S1-B), *H*[*R*_0_; ***θ***] simplifies to


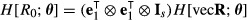
eqn 58

where **e**_1_ is the *n* × 1 vector with 1 as its first entry and zeros elsewhere.

## A case study: *Calathea ovandensis*

*Calathea ovandensis* is a neotropical perennial herb that inhabits forest understories. Horvitz & Schemske (1995) developed a stage-structured matrix model for *C. ovandensis* that contains eight stages distinguished by size and reproductive ability: seeds, nonreproductive stages (seedlings, juveniles, pre-reproductive), and reproductive stages (small, medium, large and extra large). Plants may grow larger, remain in the same size class, or shrink at each time step; larger adults are typically more fecund.

Horvitz and Schemske summarized four years of population dynamics from four plots of *C. ovandensis* with a series of 8×8 projection matrices. The average of these matrices, weighted by the observed stage abundances and transition frequencies, is given in Table 8 of Horvitz & Schemske (1995) as


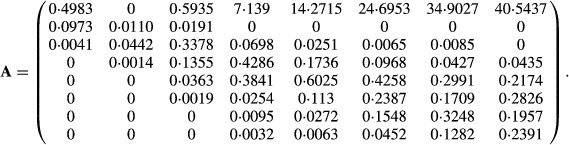
eqn 59

The dominant eigenvalue of this matrix is 0·9923, indicating a near-steady state population.

To obtain the second derivatives of λ to the entries of **A**, we calculated the Hessian *H*[λ; vec**A**] using (25). It is a symmetric 64 × 64 matrix (Fig. 1). In this example, and in others with large projection matrices, *H*[λ; vec**A**] contains many entries and may be difficult to interpret, even when entries that are fixed at 0 are omitted. Most of the second derivatives here are small in magnitude (Fig. 1b) with the exception of a few entries, including the highly negative 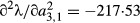 and ∂^2^λ/∂*a*_3,1_∂*a*_4,2_ = −75·64, where *a*_3,1_ is the transition probability from seed to juvenile and *a*_4,2_ is the transition probability from seedling to pre-reproductive.

Using (38), we calculated the Hessian *H*[λ; ***θ***] for a set of lower-level parameters ***θ***. For example, the stage-specific survival probabilities are lower-level parameters that affect multiple matrix entries. To analyse these using (38), write the survival probabilities in a vector ***σ***, which is given by the column sums of **U**, so that


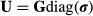
eqn 60

where **G** describes stage transitions conditional on survival (Caswell 2011). The Hessian of λ with respect to ***σ*** is given by (38), with the parameter vector **θ** given by ***σ***. Calculating this Hessian requires the first and second derivatives of **A** with respect to ***σ***. The first derivatives, assuming that **F** does not depend on ***σ*** (i.e. prebreeding census), are


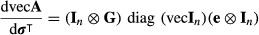
eqn 61

(see Caswell and Salguero-Gómez 2013, Appendix A).

The second derivatives of **A** are given by *H*[vec**A**; ***σ***], the derivative of (61) with respect to ***σ***. However, none of the terms in (61) depend on ***σ***, so *H*[vec**A**;***σ***] is a zero matrix. Thus, the matrix **B** in (38) reduces to


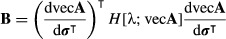
eqn 62

where 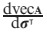 is given by (61) and *H*[λ;vec**A**] is given by (25).

**Fig. 2:**
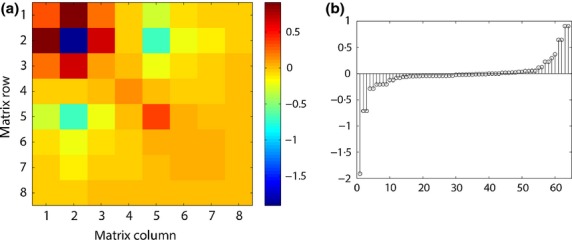
(a) The Hessian *H*[λ;***σ***], giving the second derivatives of λ with respect to stage-specific survival probabilities *σ*_*i*_, for *C. ovandensis*. (b) The Hessian entries in 2a, sorted in ascending order.

**Fig. 3:**
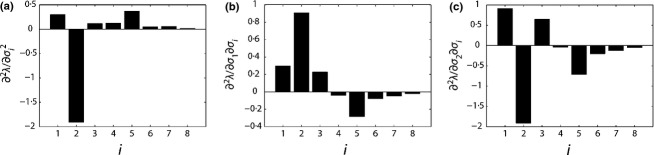
Three sets of second derivatives from *H*[λ; ***σ***] (Figure 2). (a) The pure second derivatives 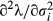. (b) The mixed second derivatives ∂^2^λ/∂*σ*_1_∂*σ*_*i*_. (c) The mixed second derivatives ∂^2^λ/∂*σ*_2_∂*σ*_*i*_.

The resulting Hessian matrix with respect to the lower-level survival probabilities, *H*[λ;***σ***], is shown in Fig. 2. These second derivatives are generally of smaller magnitude than those of *H*[λ;vec**A**] (Fig. 1). The largest second derivatives in *H*[λ;***σ***] appear in rows 1 and 2 (equivalently, columns 1 and 2). Figure 3 highlights the mixed second derivatives ∂^2^λ/∂*σ*_1_∂*σ*_*i*_ and ∂^2^λ/∂*σ*_2_∂*σ*_*i*_, along with the pure second derivatives 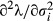.

*C. ovandensis* has several large second derivatives involving *σ*_1_ and *σ*_2_ (the first two rows or columns of Fig. 2, which are shown separately in Fig. 3b,c). As discussed in the section ‘Second-order sensitivity analysis and growth rate estimation,’ this indicates that the sensitivity of λ to stage 1 (seed) and stage 2 (seedling) survival will be especially responsive to changes in later survival. Similarly, the sensitivity of λ to later survival is especially responsive to changes in seed and seedling survival.

When interpreted in terms of selection gradients, recall from the section, ‘Characterizing nonlinear selection processes’ that selection on a single trait is concave, linear or convex if ∂^2^λ/∂θ^2^ is negative, zero or positive. Selection on two traits is negatively or positively correlational if ∂^2^λ/∂θ_1_∂θ_2_ is negative or positive. *C. ovandensis* is experiencing nearly linear selection on survival in stage 8 (∂^2^λ/∂*σ*_8_ ≈ 0), concave selection on survival in stage 2, and convex selection on survival in stages 1, 3, 4 and 5. There is negative correlational selection between survival in stage 1 (seeds) or 2 (seedlings) and survival in stages 4-8 (adults), and positive correlational selection between seed or seedling survival and survival in stages 1–3 (pre-adults). This indicates that seed and seedling survival are being selected to decrease their correlation with adult survival, but to increase their correlation with pre-adult survival.

Because the Hessian matrices include second derivatives with respect to all possible pairs of characters (matrix entries or lower-level parameters), they contain a great deal of information, and there are no established standards for displaying the results. We have shown several possibilities that may be useful: colour plots, plots that remove matrix entries that are of no interest because they are structural zeros, and plots displaying the range of magnitudes of the second derivatives. Others will no doubt be developed. The Matlab code used to generate the analysis is included in the Supporting Information.

## Sensitivity analysis of stochastic growth rates

An application in which second derivatives are not the objective, but in which the Hessian matrix plays a role, is the sensitivity of Tuljapurkar's small-noise approximation to the stochastic growth rate log λ_*s*_ (‘Section Sensitivity of the stochastic growth rate’). Tuljapurkar's approximation can be written in terms of the Jacobian matrix **D** of first derivatives of the dominant eigenvalue of the mean projection matrix, 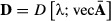. Assuming that environments are uncorrelated in time,


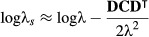
eqn 63

where **C** is the covariance matrix of the entries of the population projection matrix


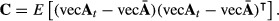
eqn 64

The sensitivity of the stochastic growth rate can be obtained by differentiating (63) with respect to the entries of 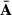. The sensitivity of the stochastic growth rate to 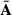, leaving the variances and covariances fixed, depends on the second derivatives of λ as


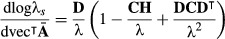
eqn 65

where 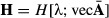 is the Hessian matrix of second derivatives. A derivation of (65) is provided in Appendix S1-C. Much more powerful and general approaches to sensitivity analysis of the stochastic growth rate are available in recent developments of the Monte Carlo method (e.g. Caswell, [Bibr b6],[Bibr b10], Tuljapurkar, Horvitz & Pascarella 2003, Haridas & Tuljapurkar 2005, Horvitz, Tuljapurkar & Pascarella 2005). This approximate result may, however, be useful in situations where the stochastic environment is defined directly in terms of the covariance matrix **C** of the vital rates.

## Discussion

Although the first derivatives of population growth rates are commonly used in ecology and demography, tools for calculating the second derivatives are not nearly as well-established, even though second derivatives also have a variety of potential applications. To this end, we have derived new, more easily computable formulae for the second derivatives of three population growth rate measures – the discrete-time growth rate λ, the continuous-time growth rate *r*, and the per-generation growth rate *R*_0_ – both with respect to projection matrix entries and to lower-level parameters. Table 2 provides an overview of the results, with directions to the equations defining the Hessian matrix, containing all second-order partial derivatives, for each type of growth rate and each type of independent variable.

**Table 2:** An overview of the formulae for the second derivatives of population growth rates (λ, *r, R*_0_) with respect to matrix entries (A, U, F), or to lower-level parameters (*θ, σ*). The equation number for the corresponding Hessian matrix is given in the third column; auxiliary equations for terms in the Hessian expressions are given in the fourth column. The Matlab functions used to calculate each Hessian, as provided in the supplemental material, are listed in the last column

Growth rate	Variables	Hessian equation	Auxiliary equations	MATLAB script
λ	**A**	(25)	(21), (22)	Hlambda_A.m
	***θ***	(38)	(25)	Hlambda_theta.m
	***σ***	(62)	(25), (61)	Hlambda_sigma.m
*r*	**A**	(43)	(25)	Hr_A.m
	***θ***	(45)	(38)	Hr_theta.m
*R*_0_	**U**	(54)	(25), (48), (53)	HR0_U.m
	**F**	(57)	(25)	HR0_F.m
	***θ***	(47)	(25), (B-2), (B-7) (or B-12/B-13), (B-8), (B-9), (B-10), (B-11)	HR0_theta.m
		(58) if one offspring type	(B-7) (or B-12/B-13), (B-8), (B-9), (B-10), (B-11)	HR0_theta_1.m

The matrix calculus approach is comprehensive, and even though the formulae may appear complicated, they are easy to apply with any matrix-oriented software. Other methods for finding second derivatives are either more limited or require more difficult and error-prone calculations. Cohen (1978), for instance, derives the second pure derivatives of λ with respect to the diagonal elements of the projection matrix (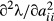) only. The approaches of Deutsch & Neumann (1984) and Kirkland & Neumann (1994) rely on the calculation of group inverses, while those of Caswell (1996) require all the eigenvalues and eigenvectors of the projection matrix. McCarthy *et al*.'s method (2008) uses transfer functions rather than eigenvectors and is more complicated when handling lower-level parameters.

Population growth rate, no matter how it is measured, is important in many ecological and evolutionary problems. It is hoped that the methods presented here will contribute to a deeper understanding of the response of growth rates to changes in parameters.

## Data accessibility

MATLAB scripts: uploaded as online supporting information

## Supporting Information

**Supplemental files S1–17.** MATLAB scripts for calculating second derivatives (as summarized in Table 2).

**Appendix S1**. A) First derivatives of **S** and **T**. B) Second derivatives of R0 to lower-level parameters: H[R_0_;***θ***]. C) Sensitivity of the stochastic growth rate. D) Commutation matrix code.
