# Supplementary material for: Calculating second derivatives of population growth rates for ecology and evolution
Source: Methods Ecol Evol. 2014 May 19;5(5):473–82. doi: 10.1111/2041-210X.12179 (PMC4358155; doi:10.1111/2041-210X.12179)
Supplement: Supplementary file 18 — Appendix S1. A) First derivatives of S and T. B) Second derivatives of R0 to lower-level parameters: H[R0;θ]. C) Sensitivity of the stochastic growth rate. D) Commutation matrix code. [file mee30005-0473-sd18.pdf]

Appendices

A First derivatives of $\mathbf{S} = \frac{d\mathbf{vec}\mathbf{A}}{d\boldsymbol{\theta}^\intercal}$ and $\mathbf{T} = \frac{d\lambda}{d\mathbf{vec}^\intercal\mathbf{A}}$

In (30), we defined

$$\mathbf{S} = \frac{d\text{vec}\mathbf{A}}{d\boldsymbol{\theta}^\intercal} \quad \text{and} \quad \mathbf{T} = \frac{d\lambda}{d\text{vec}^\intercal\mathbf{A}} = \mathbf{w}^\intercal \otimes \mathbf{v}^\intercal \ . \tag{A-1}$$

To evaluate (34), we require the first derivatives of $\mathbf{S}$ and $\mathbf{T}$. For the first derivative of $\mathbf{S}$:

$$\frac{d\text{vec}\mathbf{S}}{d\boldsymbol{\theta}^\intercal} = \frac{d}{d\boldsymbol{\theta}^\intercal}\text{vec}\left[\frac{d\text{vec}\mathbf{A}}{d\boldsymbol{\theta}^\intercal}\right] \ . \tag{A-2}$$

Apply the commutation matrix (vec-permutation matrix) $\mathbf{K}_{m,n}$, a constant matrix of 0's and 1's that can be calculated using the MATLAB function provided in Appendix D. For any $m \times n$ matrix $\mathbf{A}$, there is a unique $mn \times mn$ matrix $\mathbf{K}_{m,n}$ such that:

$$\mathbf{K}_{m,n}\text{vec}\mathbf{A} = \text{vec}(\mathbf{A}^\intercal) \ . \tag{A-3}$$

Using both the commutation matrix and the definition of the Hessian matrix in (9):

$$\frac{d\text{vec}\mathbf{S}}{d\boldsymbol{\theta}^\intercal} = \frac{d}{d\boldsymbol{\theta}^\intercal}\text{vec}\left[\frac{d\text{vec}\mathbf{A}}{d\boldsymbol{\theta}^\intercal}\right] \tag{A-4}$$

$$= \frac{d}{d\boldsymbol{\theta}^\intercal}\mathbf{K}_{s,n^2}\text{vec}\left[\left(\frac{d\text{vec}\mathbf{A}}{d\boldsymbol{\theta}^\intercal}\right)^\intercal\right] \tag{A-5}$$

$$= \mathbf{K}_{s,n^2}H\left[\text{vec}\mathbf{A};\boldsymbol{\theta}\right] \ . \tag{A-6}$$

Note that for a $m \times n$ matrix $\mathbf{X}$ and a $p \times q$ matrix $\mathbf{Y}$,

$$\mathbf{K}_{p,m}(\mathbf{X} \otimes \mathbf{Y}) = (\mathbf{Y} \otimes \mathbf{X})\mathbf{K}_{q,n} \tag{A-7}$$

and

$$\mathbf{K}_{n,1} = \mathbf{K}_{1,n} = \mathbf{I}_n \tag{A-8}$$

so (35) becomes

$$(\mathbf{I}_s \otimes \mathbf{T})\frac{d\text{vec}\mathbf{S}}{d\boldsymbol{\theta}^\intercal} = (\mathbf{I}_s \otimes \mathbf{T})\mathbf{K}_{s,n^2} H\left[\text{vec}\mathbf{A}; \boldsymbol{\theta}\right] \tag{A-9}$$

$$= (\mathbf{T} \otimes \mathbf{I}_s) H\left[\text{vec}\mathbf{A}; \boldsymbol{\theta}\right] \ . \tag{A-10}$$

To find (36), differentiate the expression for $\mathbf{T}$

$$\frac{d\text{vec}\mathbf{T}}{d\boldsymbol{\theta}^\intercal} = \frac{d}{d\boldsymbol{\theta}^\intercal}\text{vec}\left[\frac{d\lambda}{d\text{vec}\mathbf{A}^\intercal}\right] \tag{A-11}$$

and use the chain rule to rewrite it in terms of the derivative with respect to $\mathbf{A}$

$$\frac{d\text{vec}\mathbf{T}}{d\boldsymbol{\theta}^\intercal} = \frac{d}{d\text{vec}\mathbf{A}^\intercal}\text{vec}\left[\frac{d\lambda}{d\text{vec}\mathbf{A}^\intercal}\right]\frac{d\text{vec}\mathbf{A}}{d\boldsymbol{\theta}^\intercal} \ . \tag{A-12}$$

The first part of (A-12) is equivalent to the Hessian expression (9), so:

$$\frac{d\text{vec}\mathbf{T}}{d\boldsymbol{\theta}^\intercal} = H\left[\lambda; \text{vec}\mathbf{A}\right]\mathbf{S} \ . \tag{A-13}$$

B Second derivatives of R_0 to lower-level parameters: $H\left[R_0; \boldsymbol{\theta}\right]$

In Section 3.3, we calculated the second derivatives of the net reproductive rate R_0 with respect to $\mathbf{U}$ and $\mathbf{F}$. Here, we present the fully general second derivatives of R_0 with respect to a parameter vector $\boldsymbol{\theta}$.

As written in (47), the general expression for the Hessian of R_0 with respect to $\boldsymbol{\theta}$ is

$$H\left[R_0; \boldsymbol{\theta}\right] = \frac{1}{2}\left(\mathbf{B} + \mathbf{B}^\intercal\right) \tag{B-1a}$$

where

$$\mathbf{B} = (\mathbf{w}_R^\intercal \otimes \mathbf{v}_R^\intercal \otimes \mathbf{I}_s) H\left[\mathrm{vec}\mathbf{R}; \boldsymbol{\theta}\right] + \left(\frac{d\mathrm{vec}\mathbf{R}}{d\boldsymbol{\theta}^\intercal}\right)^\intercal H\left[R_0; \mathrm{vec}\mathbf{R}\right] \frac{d\mathrm{vec}\mathbf{R}}{d\boldsymbol{\theta}^\intercal} \tag{B-1b}$$

and $\mathbf{w}_R$ and $\mathbf{v}_R$ are the right and left eigenvectors of $\mathbf{R}$. As noted in the main text, evaluating $\mathbf{B}$ requires the second derivatives of R_0 with respect to $\mathbf{R}$ (the Hessian $H\left[R_0; \mathrm{vec}\mathbf{R}\right]$, which is given by (25) using the dominant eigenvalues and eigenvectors of $\mathbf{R}$ rather than those of $\mathbf{A}$), and the first and second derivatives of $\mathbf{R}$ with respect to $\boldsymbol{\theta}$. We will now calculate these derivatives of $\mathbf{R}$ with respect to $\boldsymbol{\theta}$.

The first derivatives of $\mathbf{R}$ with respect to $\boldsymbol{\theta}$ are

$$\frac{d\mathrm{vec}\mathbf{R}}{d\boldsymbol{\theta}^\intercal} = (\mathbf{N}^\intercal \otimes \mathbf{I}_n)\frac{d\mathrm{vec}\mathbf{F}}{d\boldsymbol{\theta}^\intercal} + (\mathbf{N}^\intercal \otimes \mathbf{R})\frac{d\mathrm{vec}\mathbf{U}}{d\boldsymbol{\theta}^\intercal} \ . \tag{B-2}$$

The second derivatives of $\mathbf{R}$ with respect to $\boldsymbol{\theta}$ are given by the definition of the Hessian matrix (9):

$$H\left[\mathrm{vec}\mathbf{R}; \boldsymbol{\theta}\right] = \frac{d}{d\boldsymbol{\theta}^\intercal}\mathrm{vec}\left[\left(\frac{d\mathrm{vec}\mathbf{R}}{d\boldsymbol{\theta}^\intercal}\right)^\intercal\right] \tag{B-3}$$

$$= \frac{d}{d\boldsymbol{\theta}^\intercal}\mathrm{vec}\left[\left(\frac{d\mathrm{vec}\mathbf{F}}{d\boldsymbol{\theta}^\intercal}\right)^\intercal (\mathbf{N} \otimes \mathbf{I}_n) + \left(\frac{d\mathrm{vec}\mathbf{U}}{d\boldsymbol{\theta}^\intercal}\right)^\intercal (\mathbf{N} \otimes \mathbf{R}^\intercal)\right] \ . \tag{B-4}$$

To evaluate this expression, we will use the following rule (Magnus 2010) for derivatives of matrix products: given matrices $\mathbf{Y}$ $(n \times v)$ and $\mathbf{X}$ $(m \times n)$, the derivative of their product with respect to a third matrix $\mathbf{Z}$ $(p \times q)$ is

$$\frac{d\mathrm{vec}(\mathbf{XY})}{d(\mathrm{vec}\mathbf{Z})^\intercal} = (\mathbf{Y}^\intercal \otimes \mathbf{I}_m)\frac{d\mathrm{vec}\mathbf{X}}{d(\mathrm{vec}\mathbf{Z})^\intercal} + (\mathbf{I}_v \otimes \mathbf{X})\frac{d\mathrm{vec}\mathbf{Y}}{d(\mathrm{vec}\mathbf{Z})^\intercal} \ . \tag{B-5}$$

Applying this product rule for matrix derivatives to (B-4) gives

$$H\left[\text{vec}\mathbf{R};\boldsymbol{\theta}\right]=\left[\left(\mathbf{N}\otimes\mathbf{I}_n\right)^{\intercal}\otimes\mathbf{I}_s\right]\frac{d}{d\boldsymbol{\theta}^{\intercal}}\text{vec}\left[\left(\frac{d\text{vec}\mathbf{F}}{d\boldsymbol{\theta}^{\intercal}}\right)^{\intercal}\right]+\left[\mathbf{I}_{n^2}\otimes\left(\frac{d\text{vec}\mathbf{F}}{d\boldsymbol{\theta}^{\intercal}}\right)^{\intercal}\right]\frac{d}{d\boldsymbol{\theta}^{\intercal}}\text{vec}\left(\mathbf{N}\otimes\mathbf{I}_n\right)$$
$$+\left[\left(\mathbf{N}\otimes\mathbf{R}^{\intercal}\right)^{\intercal}\otimes\mathbf{I}_s\right]\frac{d}{d\boldsymbol{\theta}^{\intercal}}\text{vec}\left[\left(\frac{d\text{vec}\mathbf{U}}{d\boldsymbol{\theta}^{\intercal}}\right)^{\intercal}\right]+\left[\mathbf{I}_{n^2}\otimes\left(\frac{d\text{vec}\mathbf{U}}{d\boldsymbol{\theta}^{\intercal}}\right)^{\intercal}\right]\frac{d}{d\boldsymbol{\theta}^{\intercal}}\text{vec}\left(\mathbf{N}\otimes\mathbf{R}^{\intercal}\right) \tag{B-6}$$

and recognizing Hessian matrices of the form (9), we obtain

$$H\left[\text{vec}\mathbf{R};\boldsymbol{\theta}\right]=\left(\mathbf{N}^{\intercal}\otimes\mathbf{I}_{ns}\right)H\left[\text{vec}\mathbf{F};\boldsymbol{\theta}\right]+\left[\mathbf{I}_{n^2}\otimes\left(\frac{d\text{vec}\mathbf{F}}{d\boldsymbol{\theta}^{\intercal}}\right)^{\intercal}\right]\frac{d}{d\boldsymbol{\theta}^{\intercal}}\text{vec}\left(\mathbf{N}\otimes\mathbf{I}_n\right)$$
$$+\left(\mathbf{N}^{\intercal}\otimes\mathbf{R}\otimes\mathbf{I}_s\right)H\left[\text{vec}\mathbf{U};\boldsymbol{\theta}\right]+\left[\mathbf{I}_{n^2}\otimes\left(\frac{d\text{vec}\mathbf{U}}{d\boldsymbol{\theta}^{\intercal}}\right)^{\intercal}\right]\frac{d}{d\boldsymbol{\theta}^{\intercal}}\text{vec}\left(\mathbf{N}\otimes\mathbf{R}^{\intercal}\right). \tag{B-7}$$

Applying (51), the derivatives of Kronecker products appearing in (B-7) are

$$\frac{d}{d\boldsymbol{\theta}^{\intercal}}\text{vec}\left(\mathbf{N}\otimes\mathbf{I}_n\right)=\left(\mathbf{I}_n\otimes\mathbf{K}_{n,n}\otimes\mathbf{I}_n\right)\left(\mathbf{I}_{n^2}\otimes\text{vec}\left[\mathbf{I}_n\right]\right)\frac{d\text{vec}\mathbf{N}}{d\boldsymbol{\theta}^{\intercal}} \tag{B-8}$$

$$\frac{d}{d\boldsymbol{\theta}^{\intercal}}\text{vec}\left(\mathbf{N}\otimes\mathbf{R}^{\intercal}\right)=\left(\mathbf{I}_n\otimes\mathbf{K}_{n,n}\otimes\mathbf{I}_n\right)\left[\left(\mathbf{I}_{n^2}\otimes\text{vec}\left[\mathbf{R}^{\intercal}\right]\right)\frac{d\text{vec}\mathbf{N}}{d\boldsymbol{\theta}^{\intercal}}+\left(\text{vec}\mathbf{N}\otimes\mathbf{I}_{n^2}\right)\frac{d\text{vec}\mathbf{R}^{\intercal}}{d\boldsymbol{\theta}^{\intercal}}\right] \tag{B-9}$$

and note that

$$\frac{d\text{vec}\mathbf{N}}{d\boldsymbol{\theta}^{\intercal}}=\left(\mathbf{N}^{\intercal}\otimes\mathbf{N}\right)\frac{d\text{vec}\mathbf{U}}{d\boldsymbol{\theta}^{\intercal}} \tag{B-10}$$

$$\frac{d\text{vec}\mathbf{R}^{\intercal}}{d\boldsymbol{\theta}^{\intercal}}=\mathbf{K}_{n,n}\frac{d\text{vec}\mathbf{R}}{d\boldsymbol{\theta}^{\intercal}} \tag{B-11}$$

where $\frac{d\text{vec}\mathbf{R}}{d\boldsymbol{\theta}^{\intercal}}$ is given by (B-2). Substitute (B-10) and (B-11) into (B-8) and (B-9), then back into (B-7) to obtain $H\left[\text{vec}\mathbf{R};\boldsymbol{\theta}\right]$.

While the expression for $H\left[\text{vec}\mathbf{R};\boldsymbol{\theta}\right]$ may appear complicated, note that it simplifies considerably if only one of $\mathbf{U}$ or $\mathbf{F}$ is a function of $\boldsymbol{\theta}$, so that either $\frac{d\text{vec}\mathbf{F}}{d\boldsymbol{\theta}^{\intercal}}$ or $\frac{d\text{vec}\mathbf{U}}{d\boldsymbol{\theta}^{\intercal}}$ is a matrix of zeros (i.e., the parameters of interest affect only fertility or transitions, but not both).

If only $\mathbf{U}$ depends on $\boldsymbol{\theta}$:

$$H\left[\text{vec}\mathbf{R};\boldsymbol{\theta}\right] = \left(\mathbf{N}^{\intercal}\otimes\mathbf{R}\otimes\mathbf{I}_s\right)H\left[\text{vec}\mathbf{U};\boldsymbol{\theta}\right] + \left[\mathbf{I}_{n^2}\otimes\left(\frac{d\text{vec}\mathbf{U}}{d\boldsymbol{\theta}^{\intercal}}\right)^{\intercal}\right]\frac{d}{d\boldsymbol{\theta}^{\intercal}}\text{vec}\left(\mathbf{N}\otimes\mathbf{R}^{\intercal}\right). \tag{B-12}$$

If only $\mathbf{F}$ depends on $\boldsymbol{\theta}$:

$$H\left[\text{vec}\mathbf{R};\boldsymbol{\theta}\right] = \left(\mathbf{N}^{\intercal}\otimes\mathbf{I}_{ns}\right)H\left[\text{vec}\mathbf{F};\boldsymbol{\theta}\right]. \tag{B-13}$$

Ultimately, substitute $H\left[\text{vec}\mathbf{R};\boldsymbol{\theta}\right]$ as given by (B-7) (or B-12 and B-13 if appropriate), and $\frac{d\text{vec}\mathbf{R}}{d\boldsymbol{\theta}^{\intercal}}$ as given by (B-2), back into (B-1) to obtain $H\left[R_0;\boldsymbol{\theta}\right]$.

B.1 Life cycles with only one type of offspring

In the common case where there is only one type of offspring (stage 1), the expression for $H\left[R_0;\boldsymbol{\theta}\right]$ simplifies considerably. In this case, the fertility matrix $\mathbf{F}$ has nonzero entries only in its first row, and $\mathbf{R} = \mathbf{FN}$ is an upper triangular matrix. Then R_0, the dominant eigenvalue of $\mathbf{R}$, is the (1,1) entry of $\mathbf{R}$:

$$R_0 = \mathbf{e}_1^{\intercal}(\mathbf{R})\mathbf{e}_1 \tag{B-14}$$

where $\mathbf{e}_1$ is the $n\times 1$ vector with 1 as its first entry and zeros elsewhere (Caswell 2009).

The first differential of R_0 is then

$$dR_0 = \mathbf{e}_1^{\intercal}(d\mathbf{R})\mathbf{e}_1 \tag{B-15}$$

$$= \left(\mathbf{e}_1^{\intercal}\otimes\mathbf{e}_1^{\intercal}\right)d\text{vec}\mathbf{R}\,. \tag{B-16}$$

By the first identification theorem (10),

$$\frac{dR_0}{d\boldsymbol{\theta}^{\intercal}} = \left(\mathbf{e}_1^{\intercal}\otimes\mathbf{e}_1^{\intercal}\right)\frac{d\text{vec}\mathbf{R}}{d\boldsymbol{\theta}^{\intercal}} \tag{B-17}$$

571 and by the definition of the Hessian matrix (9):

$$H\left[R_0;\boldsymbol{\theta}\right] = \frac{d}{d\boldsymbol{\theta}^{\intercal}}\text{vec}\left[\left(\frac{dR_0}{d\boldsymbol{\theta}^{\intercal}}\right)^{\intercal}\right] \tag{B-18}$$

$$= \frac{d}{d\boldsymbol{\theta}^{\intercal}}\text{vec}\left[\left(\frac{d\text{vec}\mathbf{R}}{d\boldsymbol{\theta}^{\intercal}}\right)^{\intercal}\left(\mathbf{e}_1\otimes\mathbf{e}_1\right)\right] . \tag{B-19}$$

572 Applying the product rule for matrix derivatives in (B-5) to (B-19),

$$H\left[R_0;\boldsymbol{\theta}\right] = \left(\mathbf{e}_1^{\intercal}\otimes\mathbf{e}_1^{\intercal}\otimes\mathbf{I}_s\right)\frac{d}{d\boldsymbol{\theta}^{\intercal}}\text{vec}\left[\left(\frac{d\text{vec}\mathbf{R}}{d\boldsymbol{\theta}^{\intercal}}\right)^{\intercal}\right] \tag{B-20}$$

$$= \left(\mathbf{e}_1^{\intercal}\otimes\mathbf{e}_1^{\intercal}\otimes\mathbf{I}_s\right)H\left[\text{vec}\mathbf{R};\boldsymbol{\theta}\right] \tag{B-21}$$

573 where $H\left[\text{vec}\mathbf{R};\boldsymbol{\theta}\right]$ is given by (B-7) (or B-12 and B-13 if appropriate).

574 C Sensitivity of the stochastic growth rate

575 As shown in Caswell (2001), Tuljapurkar's (1982) approximation for the stochastic growth rate can
576 be written as

577 $$\log\lambda_s \approx \log\lambda - \frac{\mathbf{DCD}^{\intercal}}{2\lambda^2} \tag{C-1}$$

578 where $\mathbf{C}$ is the symmetric covariance matrix for the population projection matrix and $\mathbf{D}$ is the
579 Jacobian matrix (in this case, a row vector) for the dominant eigenvalue λ of the mean population
580 matrix $\bar{\mathbf{A}}$:

$$\mathbf{D} = D\left[\lambda;\text{vec}\bar{\mathbf{A}}\right]$$

$$= \frac{\partial\lambda}{\partial\text{vec}^{\intercal}\bar{\mathbf{A}}} . \tag{C-2}$$

581 To find the sensitivity of the stochastic growth rate to $\bar{\mathbf{A}}$, differentiate (C-1) with respect to

582 $\text{vec}^\intercal \bar{\mathbf{A}}$:

$$\frac{\partial \log \lambda_s}{\partial \text{vec}^\intercal \bar{\mathbf{A}}} = \frac{\mathbf{D}}{\lambda} + \frac{\mathbf{D}}{\lambda^3}\left(\mathbf{D}\mathbf{C}\mathbf{D}^\intercal\right) - \frac{1}{2\lambda^2}\frac{\partial\left(\mathbf{D}\mathbf{C}\mathbf{D}^\intercal\right)}{\partial \text{vec}^\intercal \bar{\mathbf{A}}} \tag{C-3}$$

$$= \frac{\mathbf{D}}{\lambda}\left(1 + \frac{\mathbf{D}\mathbf{C}\mathbf{D}^\intercal}{\lambda^2}\right) - \frac{1}{2\lambda^2}\frac{\partial\left(\mathbf{D}\mathbf{C}\mathbf{D}^\intercal\right)}{\partial \text{vec}^\intercal \bar{\mathbf{A}}} \,. \tag{C-4}$$

583 To evaluate the derivative of $\mathbf{D}\mathbf{C}\mathbf{D}^\intercal$ with respect to $\bar{\mathbf{A}}$ in the second term, we will apply the
584 product rule for vector derivatives (Magnus 2010). Given vectors $\mathbf{y}$ $(n \times 1)$ and $\mathbf{x}$ $(1 \times n)$, the
585 derivative of their product with respect to a third vector $\mathbf{z}$ is

586
$$\frac{d\text{vec}(\mathbf{x}\mathbf{y})}{d\text{vec}^\intercal \mathbf{z}} = \mathbf{y}^\intercal \frac{d\text{vec}\mathbf{x}}{d\text{vec}^\intercal \mathbf{z}} + \mathbf{x}\frac{d\text{vec}\mathbf{y}}{d\text{vec}^\intercal \mathbf{z}} \,. \tag{C-5}$$

587 Because $\mathbf{D}\mathbf{C}\mathbf{D}^\intercal$ is a scalar, $\text{vec}\left(\mathbf{D}\mathbf{C}\mathbf{D}^\intercal\right) = \mathbf{D}\mathbf{C}\mathbf{D}^\intercal$. Thus

588
$$\frac{\partial\left(\mathbf{D}\mathbf{C}\mathbf{D}^\intercal\right)}{\partial \text{vec}^\intercal \bar{\mathbf{A}}} = \mathbf{D}\mathbf{C}^\intercal \frac{\partial \text{vec}\mathbf{D}}{\partial \text{vec}^\intercal \bar{\mathbf{A}}} + \mathbf{D}\mathbf{C}\frac{\partial \text{vec}(\mathbf{D}^\intercal)}{\partial \text{vec}^\intercal \bar{\mathbf{A}}} \,. \tag{C-6}$$

589 Note that the Hessian of λ with respect to $\bar{\mathbf{A}}$ is

$$H\left[\lambda; \text{vec}\bar{\mathbf{A}}\right] = \frac{d}{d\text{vec}^\intercal \bar{\mathbf{A}}}\text{vec}\left[\left(\frac{d\lambda}{d\text{vec}^\intercal \bar{\mathbf{A}}}\right)^\intercal\right] \tag{C-7}$$

$$= \frac{\partial \text{vec}(\mathbf{D}^\intercal)}{\partial \text{vec}^\intercal \bar{\mathbf{A}}} = \frac{\partial \text{vec}(\mathbf{D})}{\partial \text{vec}^\intercal \bar{\mathbf{A}}} \,. \tag{C-8}$$

590 Letting $\mathbf{H} = H\left[\lambda; \text{vec}\bar{\mathbf{A}}\right]$ and noting that $\mathbf{C}$ is a symmetric matrix, (C-6) simplifies to

$$\frac{\partial\left(\mathbf{D}\mathbf{C}\mathbf{D}^\intercal\right)}{\partial \text{vec}^\intercal \bar{\mathbf{A}}} = \mathbf{D}\mathbf{C}^\intercal\mathbf{H} + \mathbf{D}\mathbf{C}\mathbf{H} \tag{C-9}$$

$$= 2\mathbf{D}\mathbf{C}\mathbf{H} \,. \tag{C-10}$$

591 Substituting (C-10) into (C-4), we see that the sensitivity of the stochastic growth rate with

respect to $\bar{\mathbf{A}}$ is

$$\frac{\partial \log \lambda_s}{\partial \mathrm{vec}^\intercal \bar{\mathbf{A}}} = \frac{\mathbf{D}}{\lambda}\left(1 + \frac{\mathbf{DCD}^\intercal}{\lambda^2}\right) - \frac{\mathbf{DCH}}{\lambda^2} \tag{C-11}$$

$$= \frac{\mathbf{D}}{\lambda}\left(1 - \frac{\mathbf{CH}}{\lambda} + \frac{\mathbf{DCD}^\intercal}{\lambda^2}\right) . \tag{C-12}$$

D Commutation matrix code

The following MATLAB code calculates the commutation matrix $\mathbf{K}_{m,n}$:

```
% Calculates the commutation matrix K_(m,n) such that:
% K_(m,n)*vec(A) = vec(A') where A is dimension m by n
%
% Based on http://m.feng.li/r-tips/r-commutation-matrix
function K = Kmn(m,n)

K = zeros(m*n, m*n);
m0 = 1:(m*n);

N = reshape(m0, m,n)';
n0 = N(:);

for i = 1:(m*n)
    K(m0(i), n0(i)) = 1;
end
```
